# Detecting SNPs underlying domestication-related traits in soybean

**DOI:** 10.1186/s12870-014-0251-1

**Published:** 2014-09-26

**Authors:** Ying-Hui Li, Jochen C Reif, Scott A Jackson, Yan-Song Ma, Ru-Zhen Chang, Li-Juan Qiu

**Affiliations:** The National Key Facility for Crop Gene Resources and Genetic Improvement (NFCRI)/Key Lab of Germplasm Utilization (MOA), Institute of Crop Science, Chinese Academy of Agricultural Sciences, 100081 Beijing, P.R. China; Department of Cytogenetics and Genome Analysis, Leibniz Institute of Plant Genetics and Crop Plant Research (IPK), Gatersleben, Germany; Center for Applied Genetic Technologies, University of Georgia, Athens, GA 30602 USA; Soybean Research Institute, Heilongjiang Academy of Agricultural Sciences, 150086 Harbin, China

**Keywords:** Soybean, Interspecific differentiation, Outliers, Selection, Testa color

## Abstract

**Background:**

Cultivated soybean (*Glycine max*) experienced a severe genetic bottleneck during its domestication and a further loss in diversity during its subsequent selection. Here, a panel of 65 wild (*G. soja*) and 353 cultivated accessions was genotyped at 552 single-nucleotide polymorphism loci to search for signals of selection during and after domestication.

**Results:**

The wild and cultivated populations were well differentiated from one another. Application of the F_st_ outlier test revealed 64 loci showing evidence for selection. Of these, 35 related to selection during domestication, while the other 29 likely gradually became monomorphic as a result of prolonged selection during post domestication. Two of the SNP locus outliers were associated with testa color.

**Conclusions:**

Identifying genes controlling domestication-related traits is important for maintaining the diversity of crops. SNP locus outliers detected by a combined forward genetics and population genetics approach can provide markers with utility for the conservation of wild accessions and for trait improvement in the cultivated genepool.

**Electronic supplementary material:**

The online version of this article (doi:10.1186/s12870-014-0251-1) contains supplementary material, which is available to authorized users.

## Background

The domestication of plants has been a key driver of the development of human civilization [1,2]. The necessary changes to plant phenotype and physiology have been brought about by a process of selection at key so-called “domestication” genes [1]. Both top-down and bottom-up approaches have been taken to identify the genomic regions most clearly affected by domestication and selection [3]. The former aims to isolate the genes or quantitative trait loci (QTL) responsible for a given phenotype, and has been successful in identifying a number of major effect genes in rice [4-9], maize [10,11] and wheat [12,13]. Bottom-up approaches apply population genetics strategics in which the focus is to uncover genes showing evidence for selection, followed up by attempting to link these genes to relevant phenotypes using a bioinformatics or a reverse genetics approach. Evidence for selective sweeps has been discovered in maize, rice, wheat, soybean and sunflower [2,14-19].

Bottom-up approaches can be based on either a whole-genome re-sequencing program [19-23] or by concentrating on a pre-selected set of candidate genes [2,24-26]. The whole-genome approach is powerful, but scale-up requires a major investment. Typically, the number of accessions targeted for re-sequencing is less than 35 [19-23], a figure which reduces the detection power and simultaneously increases the risk of false positives [27,28]. In addition, many of the SNPs identified by re-sequencing do not in reality signal selection, but rather are the outcome of “genetic hitchhiking” [29,30]. To overcome this problem, the “outlier scan” test has been elaborated; this widely exploited test permits the screening of a large number of accessions [31,32]. To date, “F_st_ outliers” diagnostic of selection have been informative in several plant species, including sunflower [33], maize [16,34], white spruce [26], and other conifers [35].

In soybean (*Glycine max* (L.) Merr.), the traits most closely associated with domestication are a marked increase in the size of the inflorescences and in grain yield per plant, and an enhanced level of apical dominance. Other traits that likely have been subjected to prolonged selection are the loss of testa color and increased resistance against a range of pathogens and insects. As in most crops, the effect of domestication and subsequent anthropogenic selection pressure has been to gradually reduce the genetic diversity remaining in the pool of cultivated materials. Cultivated soybean was likely domesticated from *G. soja* Sieb. & Zucc [36]. The existence of a genetic bottleneck has been established through an analysis of allelic diversity at both microsatellite and SNP loci as well as within genic sequences [37-39]. Here, an attempt was made to apply SNP genotyping to a panel of both cultivated and wild accessions to identify signals of selection, with a particular focus on testa color. The analyses reveal that combining a population genetics with a forward genetic approach provides an effective method to identify sequences that underlie an agronomic trait.

## Methods

### Plant material

**Figure 1:**
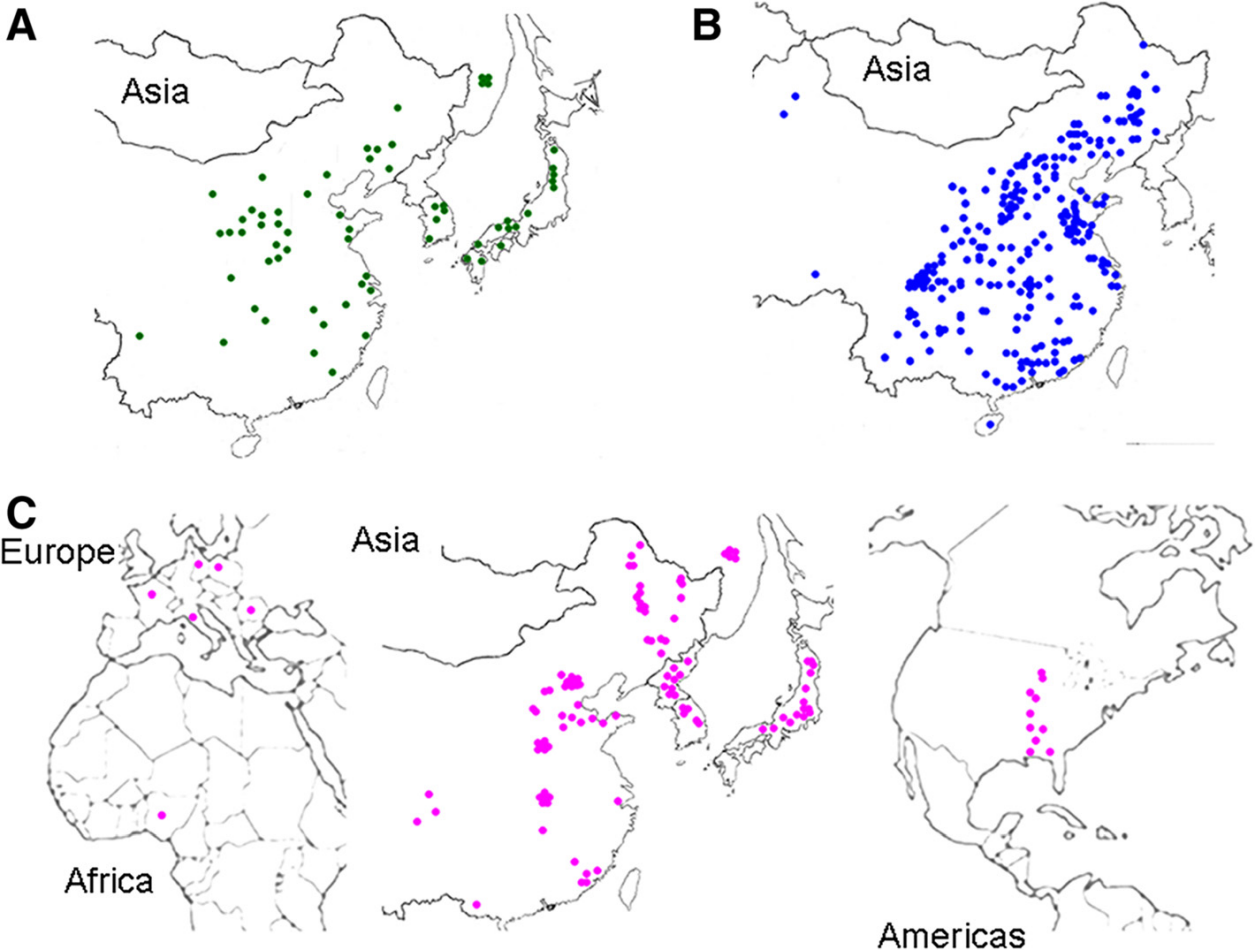
**The provenance of accessions of** ***G. max*** **and** ***G. soja*** **which formed the germplasm panel. (A)** *G. soja*. **(B)** *G. max* landraces. **(C)** *G. max* modern cultivars.

The germplasm panel comprised 65 accessions of *G. soja* and 353 of *G. max*. The provenance of the former included locations within the proposed area where soybean was domesticated (Figure 1). The cultivated population comprised 238 landraces and 115 modern cultivars. A diversity analysis of all of the wild accessions, 233 of the landraces and 65 of the modern cultivars has been reported elsewhere [37]. The additional 55 landraces and modern cultivars originated from 12 countries and were included to broaden the level of geographic representation (Additional file 1).

### Data acquisition

The allelic constitution of 363 of the 418 accessions at 554 SNP loci has been published previously [37], and these were supplemented by equivalent data for 552 of the 554 loci with respect to the 55 added accessions (Additional file 2); the data were obtained using the Illumina GoldenGate platform [40]. The GenCall and GenTrain score thresholds were set at, respectively, 80% and 0.6, as described elsewhere [37]. On average, each accession harbored 2.1% missing data (range of 0–13.9%). According to the soybean reference genome (http://www.phytozome.net) [41], the 552 SNP loci are dispersed throughout the genome, with 505 (91.5%) residing within genic DNA. About 38% of the genic SNPs lie within coding sequence, and 137 of the alleles at these loci are non-synonymous. Testa color scores for the Chinese germplasm were recovered from the Chinese soybean germplasm catalog and various other sources [42-44], while the remainder were obtained from the Germplasm Resources Information Network (USDA) database (http://www.ars-grin.gov/npgs/). Testa color was considered as a qualitative trait, with five possible states: yellow, black, brown, cyan and double, following the conventional system [45].

### Analyses of molecular diversity

Summary statistics, including the proportion of heterozygosity in the population, Nei’s indices of gene diversity and the frequency of major alleles were computed using Powermarker v3.25 [46]. A phylogenetic tree was generated, based on a neighbor-joining analysis of shared-allele distances [47] implemented in Powermarker v3.25, and this was visualized using a routine within the MEGA v4 software package [48]. Population structure was analyzed using a Bayesian Markov Chain Monte Carlo approach implemented in the software package STRUCTURE v2.1 [49]. The admixture and independent allele frequency models were adopted, testing *K* values between 1 and 10. Five runs were performed for each value of *K*, without using previous population information. The burn-in time and replication number were consistently set to 100,000.

### Identifying signals of selection

Since the low density of SNP markers (one SNP per 2 Mbp genomic region) limited the utilization of a window-sized approach to detect loci carrying a signature of selection, the summary statistic approach fdist2 [50,51] was adopted to identify SNP locus outliers. The focus was on divergence at domestication loci, so pairwise comparisons between wild accessions and landraces, and between wild accessions and modern cultivars were made. A neutral distribution of F_st_ with 50,000 interactions at the 99% confidence level was assumed, and the significance level was set at 95%.

## Results

### Population structure and genetic differentiation

**Figure 2:**
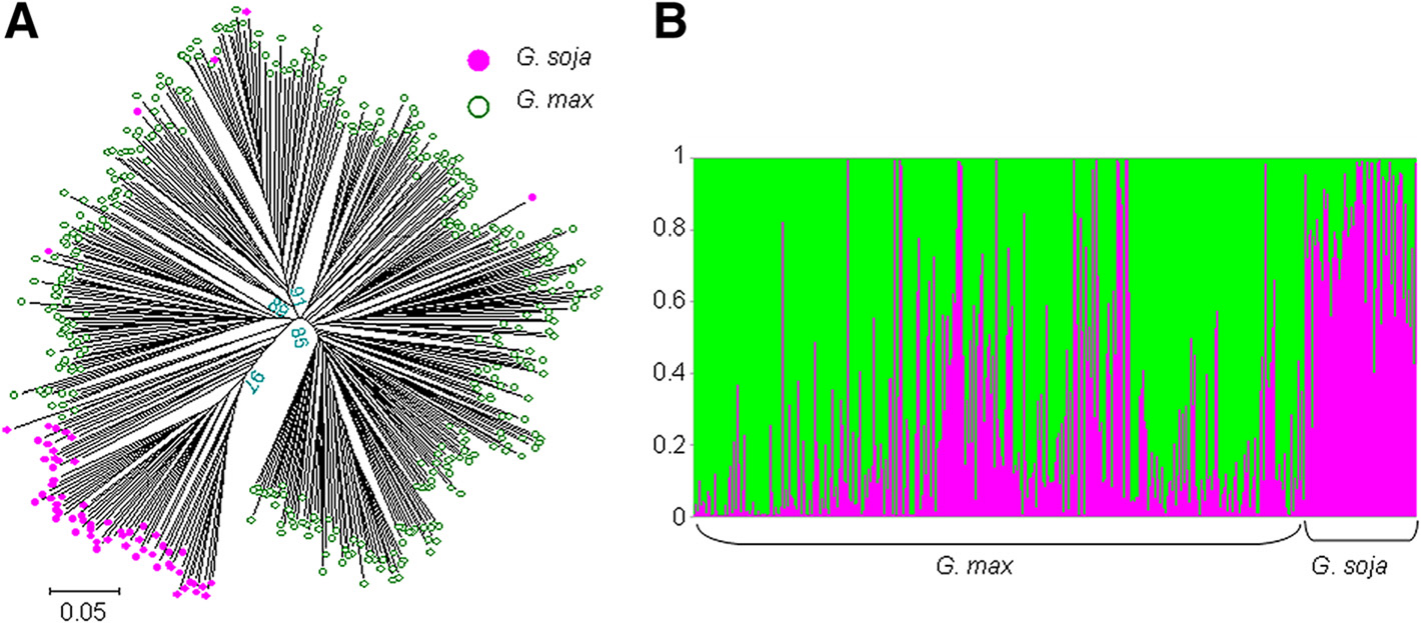
**The genetic architecture of the 418 accessions of cultivated and wild soybean. (A)** A phylogenetic tree constructed from 552 SNP loci. Pink solid circles represent *G. soja* and green hollow ones for *G. max* accessions*.* **(B)** In the STRUCTURE analysis, the groups formed at *K* = 2 correspond to *G. soja* and *G. max*. For legibility, the names of individual accessions have been omitted.

The addition of 55 accessions to the germplasm panel resulted in a slightly higher estimate of the extent of genetic diversity (Additional file 2) compared to that reported previously [39]. The population structure obtained was consistent with a discontinuity between the wild and cultivated clusters (*K* = 2), but there was evidence for introgression from wild to cultivated germplasm (Figure 2A, B). Based on mean pairwise F_st_ values, the wild, landrace and modern cultivar subsets were judged to be genetically distinct (*p* < 0.05). The extent of the differentiation was greatest between the wild and modern cultivar subsets (F_st_ = 0.162), and least between the landraces and modern cultivars (F_st_ = 0.047).

### Detection of domestication genes

**Figure 3:**
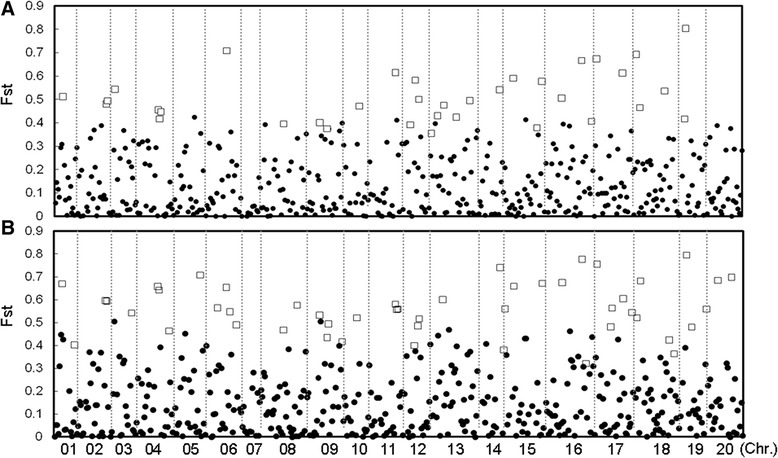
**The detection of SNP locus outliers and related F**_**st**_ **values.** The 554 loci have been ordered along the horizontal axis according to their genomic location (Additional file 2). **(A)** The 35 outlier loci identified in the comparison between wild germplasm and the landraces, **(B)** The 53 outlier loci identified in the comparison between wild germplasm and the modern cultivars. The outliers associated with a confidence level of >95% have been indicated by open squares. The vertical dotted lines separate the 20 chromosomes from one another.

**Figure 4:**
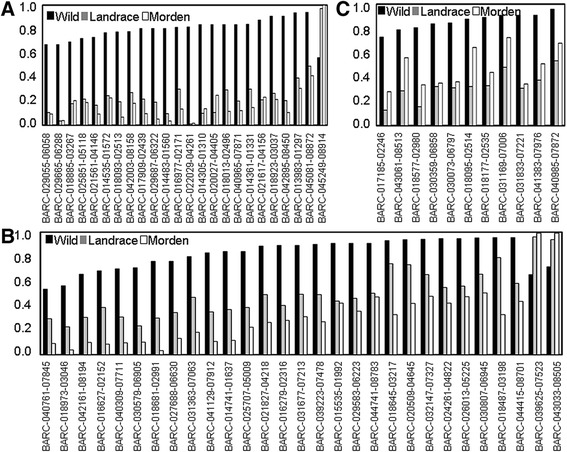
**Allele frequencies at SNP locus outliers. (A)** The 24 outliers in common between wild accessions *vs* landraces and wild accessions *vs* modern cultivars. **(B)** The 29 outliers specific to the wild accessions *vs* modern cultivars comparison. **(C)** The 11 outliers specific to the wild accessions *vs* landraces comparison.

The presence of signatures of selection during domestication was inferred by comparing the allelic status at the 552 SNP loci between the wild and the landrace subgroups (“W-LC” comparison). In all, 6.3% of the loci were identified as SNP locus outliers at the 95% confidence level (Figure 3A). The F_st_ values of the SNP locus outliers ranged from 0.36-0.80 and were 3.3-7.4 fold higher than the mean F_st_ value taken over the full set of loci (0.11). Applying the same test to the comparison between the wild accessions and the modern cultivars (“W-MC”) revealed 9.6% of the loci to be SNP locus outliers (Figure 3B). In all, nearly 70% (24/35) of the W-LC outliers were also outliers in W-MC. The major alleles of the wild population in these outlier loci changed to minor alleles in the populations of landrace or modern cultivars (Figure 4A). The strongest signal of selection was associated with the locus BARC-022029-04261, at which the major allele was represented in 82.0% of the wild accessions, but just 1.1% in the landraces and 1.7% in the modern cultivars.

In addition to the 24 shared SNP locus outliers, there were 11 W-LC- and 29 W-MC-specific ones. The frequencies of the major allele at most of the W-MC-specific loci decreased step-wise from wild accessions to landraces to modern cultivars (Figure 4B), indicating that these loci may be linked to genes/QTL subjected to prolonged selection during post domestication. The 11 W-LC-specific SNP locus outliers may represent domestication genes not subjected to selection during post domestication.

The genomic location of each of the 64 SNP locus outliers was obtained by querying the cv. Williams 82 whole genome sequence (http://www.phytozome.net) with the sequences flanking the SNP. This analysis identified regions on 19 of the 20 chromosomes (chromosome 7 had no hits). Chromosome 13 (Gm13) harbored the largest number of SNP locus outliers (six) (Additional file 3). Nine of the outliers mapped to intergenic regions. Of the 55 genic outliers, one was located in 5′-UTR, 25 in 3′-UTRs, 14 in introns and 15 (23.4%) within coding sequences. Among the latter, 13 were non-synonymous. Based on GO analysis, gene function was assignable to 38 of the W-LC + MC sequences harboring an SNP locus outlier [52]. Eleven of the genes, including five of the 13 genes harboring a non-synonymous SNP, were associated with the abiotic stress response (Additional file 3).

### Association analysis for testa color

**Figure 5:**
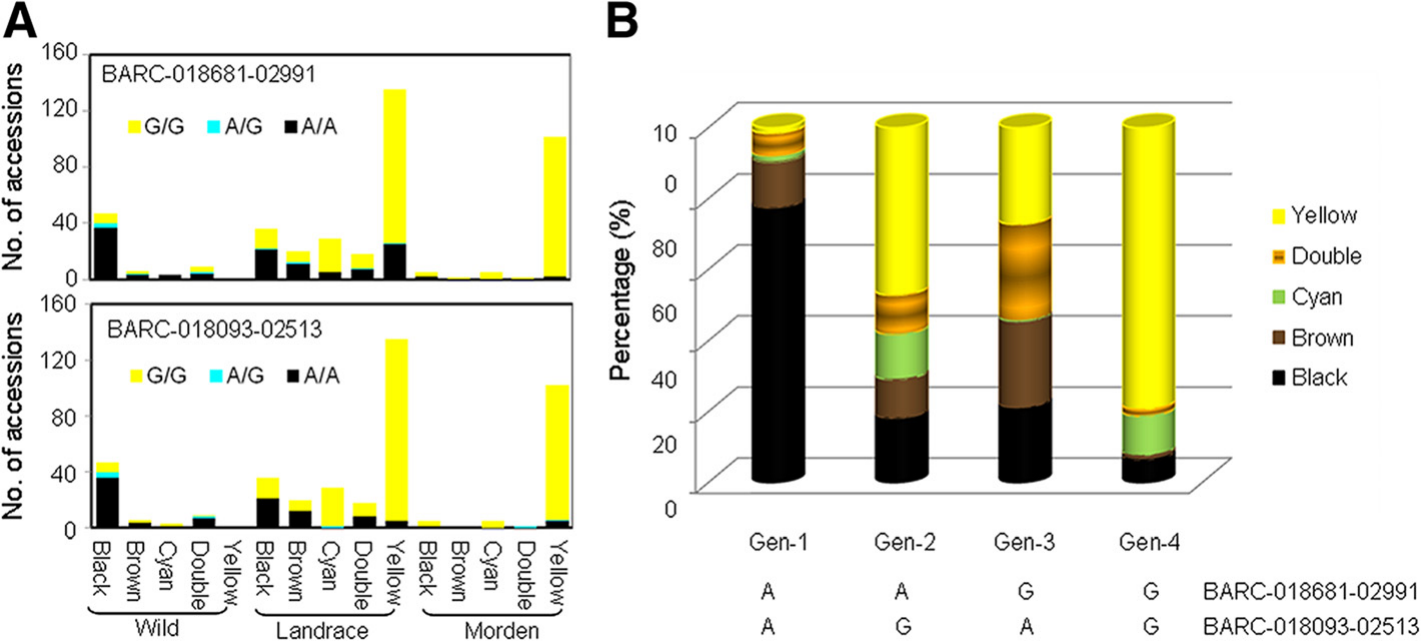
**SNP locus outliers associated with testa color. (A)** The relationship between SNP genotype and testa color in wild accessions, the landraces and the modern cultivars. **(B)** The frequency of the four haplotype classes based on SNPs within BARC-018681-02991 and BARC-018093-02513.

Most domesticated soybean materials are yellow-seeded, while black testa types predominate in wild accessions (Additional file 4). In the present germplasm panel, 47 of the 65 wild accessions were black-seeded, and 237 of the 352 domesticated ones were yellow-seeded. The yellow testa trait was more frequent in the set of modern cultivars than in the set of landraces (Additional file 4). A comparison of SNP genotype with testa color across the full set of 418 accessions identified ten SNP loci potentially linked to the trait (Additional file 5). Of these, eight were W-LC + MC SNP locus outliers and two were W-MC-specific outliers. An analysis of the distribution of testa color and SNP locus outlier allele within the three populations (wild accessions, landraces and modern cultivars) is given in Additional file 6. Allele frequencies at nine of the ten loci (the exception was BARC-045249-08914) were correlated with testa color in the wild accession and modern cultivar populations, but the correlation was only retained for two of the loci (BARC-018681-02991 and BARC-018093-02513) when all three populations were considered (Figure 5A). With respect to BARC-018681-02991, 85.1% of the black testa wild accessions and 61.1% of the black testa landraces harbored the A allele, while 81.5% of the yellow testa landraces and 98.0% of the yellow testa modern cultivars carried the G allele. Similarly at BARC-018093-02513, 85.1% of the black testa wild accessions and 58.3% of the black testa landraces harbored the A allele, while 96.3% of yellow testa landraces and 95.1% of the yellow testa modern cultivars carried the G allele. Four genotype combinations were recognized: Gen-1 (BARC-018681-02991 A, BARC-018093-02513 A), Gen-2 (AG), Gen-3 (GA), and Gen-4 (GG). The association between genotype combination and testa color was somewhat stronger than those based on a single locus: 77.0% of Gen-1 accessions were black seeded, while 79.3% of Gen-4 ones were yellow seeded (Figure 5B).

## Discussion

The strong selection pressure applied particularly during crop domestication and later subsequent genetic improvement has greatly narrowed the genetic base of cultivated types [23,53]. The current analysis identified 64 SNP locus outliers at which there was a significant difference (*P* < 0.05) in diversity between wild and cultivated soybean populations, but failed to establish any clear distinction between landraces and modern cultivars, consistent with the conclusion drawn in previous diversity studies that wild germplasm has become more strongly differentiated from landraces than landraces have become from modern cultivars [19,37,38]. A pedigree analysis has established that landraces have provided more than 76% of the nuclear genome carried by 1,300 Chinese modern cultivars released over the period 1923–2005 [54].

Even though selection signals have been identified in a number of genes, it is uncertain whether they are in reality identifying the presence of a domestication-associated genetic bottleneck [55] as opposed to reflecting the long term outcome of genetic improvement [56]. SSR markers in the vicinity of QTL underlying traits of agronomic importance tend to show a stronger level of genetic differentiation between wild and cultivated types than those unlinked to a known QTL [57]. When the location of the present SNP set was aligned with domestication-related QTL, it was established that six SNP locus outliers were linked to a domestication QTL, controlling the traits such as twining habit, maturity time, flower color, seed weight, protein content and resistance to soybean cyst nematode [58-62] (Additional file 3). In addition, eight of the SNP locus outliers are located around 1 Mbp distant from a QTL mapped in a population bred from a cross between a wild accession and a cultivated line [59-62]. Thus, it is likely that several of the SNP locus outliers identified here will have contributed to the phenotypic differentiation between wild and cultivated soybeans. SNPs BARC-025897-05144, BARC-031461- 07098 and BARC-022043-04271 used in this study are located around 1 Mbp distant from an isoflavone synthase (IFS) gene (*IFS2*, *Glyma13g24200*), which controls isoflavone accumulation and is most expressed in the developing seed in soybean [63-65]. As isoflavone was not subject to selection during domestication, we used these SNPs to evaluate whether there exists a big change to detect false positive outliers. We observed for none of the relevant SNPs significant outliers, which suggests that our study is only marginally afflicted with an inflated rate of false-positives.

Testa color in soybean is controlled by five genes, namely *I*, *T*, *W1*, *R* and *O* [66]. A screen of 170 cultivated and 102 wild accessions based on sequence variation within the testa color-associated genes encoding *f*lavonoid 3′-hydroxylase (*F3′H*) and flavonoid 3′,5′-hydroxylas*e* (*F3′5′H*) has shown that the joint allele constitution was more predictive of testa color than was the allelic state at either one of the two genes on its own [66]. Here, two SNP locus outliers (BARC-018681-02991 and BARC-018093-02513) were associated with testa color. One of the resulting four genptype combinations (Gen-4) was carried by 79.3% of the yellow testa accessions, a slightly lower proportion than was associated with a differently constituted haplotype [66]; at the same time, 77.4% of the black-seeded accessions carried Gen-1, a rather higher proportion than was recorded for the differently constituted haplotype [66].

Some of the SNP locus outliers represent potential markers for other aspects of morphological differentiation between *G. max* and *G. soja*. Cultivated soybean plants are shorter and more compact than wild soybean plants, characteristics which better fit the requirements of modern soybean production systems. One of the outliers (BARC-040965-07871) mapped within the 3′ UTR of *Glyma15g41130*, a gene which encodes a SAUR-like auxin-responsive protein family, and which is linked to a QTL controlling plant height [67]. Members of this gene family have been associated with the determination of flowering time and the regulation of growth and plant architecture [20].

## Conclusions

Genetic variation is the *sine qua non* for crop improvement. The domestication of soybean and the subsequent prolonged period of selection have resulted in a major loss in its genetic diversity. An overly narrow genetic base compromises the potential for achieving continuing gains from selection, underlining the importance of germplasm conservation, particularly of wild forms. At the same time, the identification of which genes were involved in domestication is required to recognize novel genes/alleles likely to contribute to soybean improvement. The SNP locus outliers identified here should not only aid in elaborating rational strategies for the conservation of wild germplasm [68], but may well also provide a source of markers suitable for the application of molecular breeding aimed at broadening the genetic base of soybean.

## Additional files

Additional file 1:
**The provenance of the 55 additional soybean accessions.**
Additional file 2:
**Population statistics associated with the 552 SNP loci used to genotype the 418 soybean accessions.**
Additional file 3:
**Properties of the 64 SNP locus outliers in soybean.**
Additional file 4:
**The distribution of testa color among the wild accessions, landraces and modern cultivars.**
Additional file 5:
**The joint distribution of testa color and allelic status at SNP locus outliers associated with soybean testa color.**
Additional file 6:
**The joint distribution of testa color and allele at ten SNP locus outliers among the wild accessions, landraces and modern cultivars.**
