# Supplementary figures and images for: Detecting SNPs underlying domestication-related traits in soybean
Source: BMC Plant Biol. 2014 Sep 26;14:251. doi: 10.1186/s12870-014-0251-1 (PMC4180965; doi:10.1186/s12870-014-0251-1)

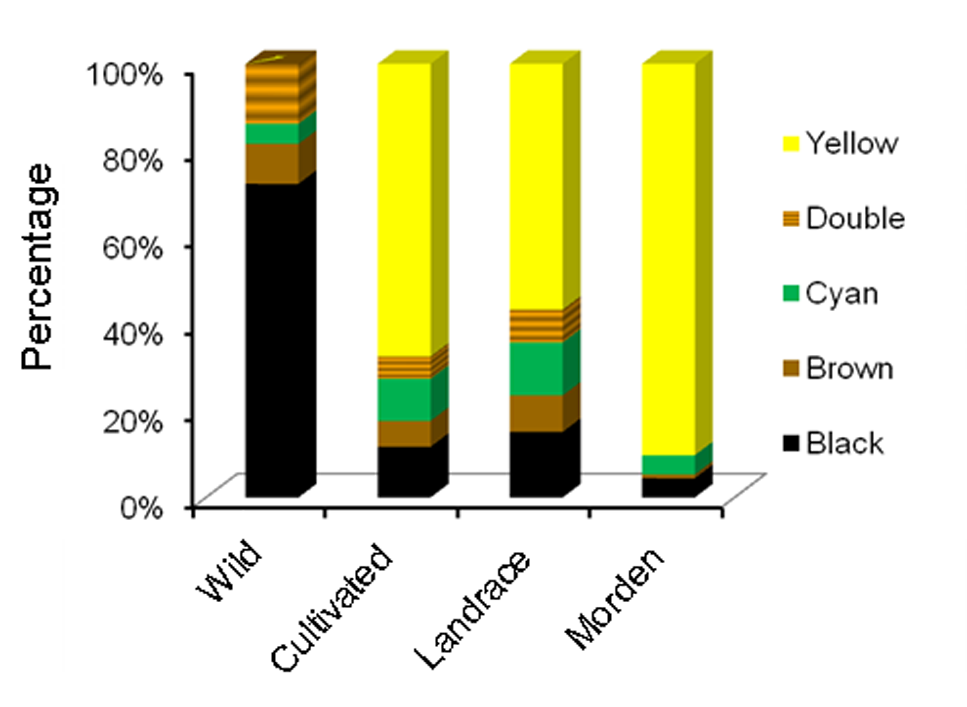

Supplement: Additional file 4: — The distribution of testa color among the wild accessions, landraces and modern cultivars. [file 12870_2014_251_MOESM4_ESM.tiff]

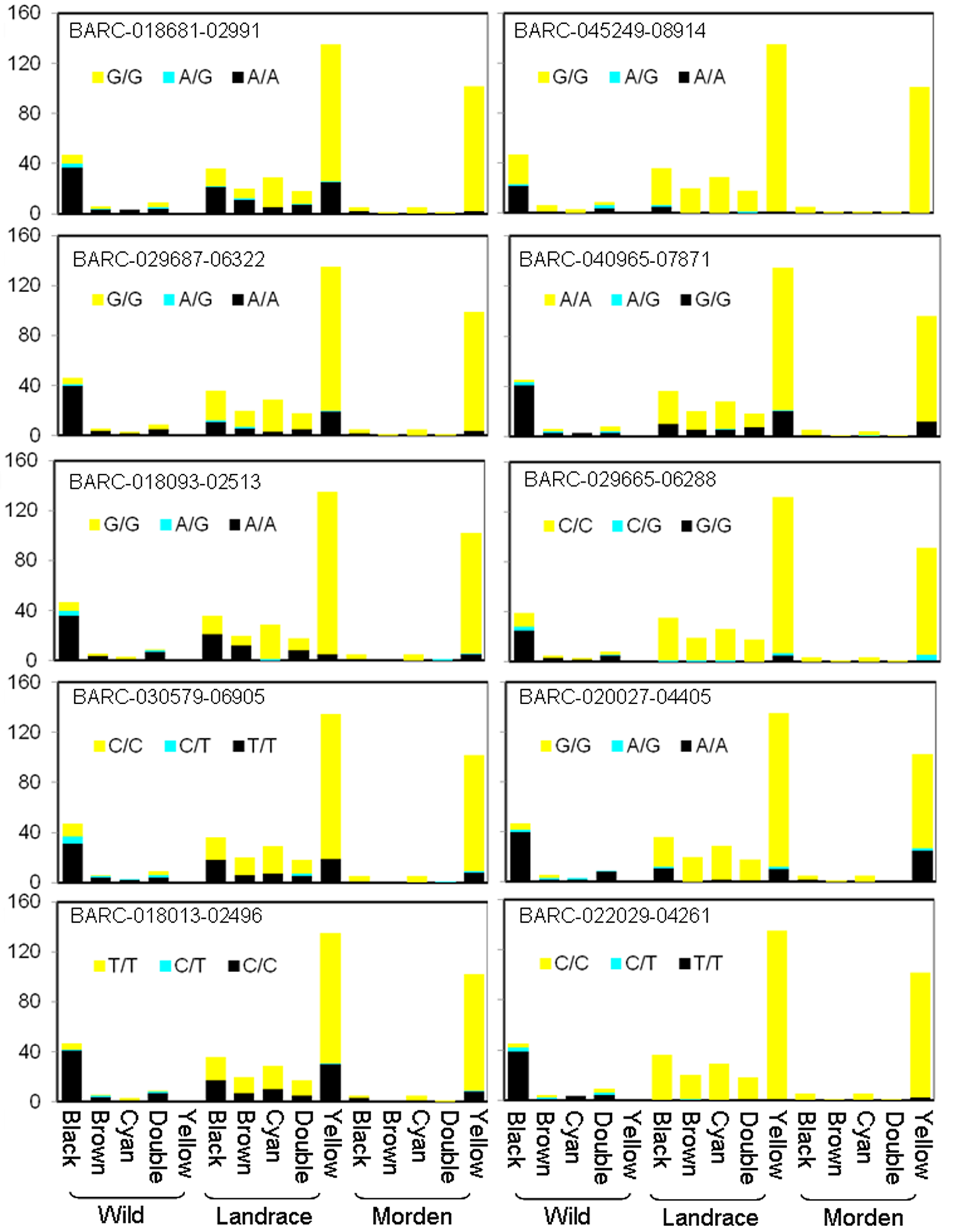

Supplement: Additional file 6: — The joint distribution of testa color and allele at ten SNP locus outliers among the wild accessions, landraces and modern cultivars. [file 12870_2014_251_MOESM6_ESM.tiff]
